# Development of Age-Friendly Health Systems in Culturally Diverse Settings

**DOI:** 10.1093/geroni/igaa057.2961

**Published:** 2020-12-16

**Authors:** Jung-Ah Lee, Lisa Gibbs, Camille Fitzpatrick, Neika Saville

**Affiliations:** 1 University of California, Irvine, Irvine, California, United States; 2 UCI Health ACO, Irvine, California, United States; 3 UC Irvine School of Nursing, Irvine, California, United States

## Abstract

UC Irvine (UCI) GWEP partnerships were designed to address a startling increase in impoverished older adults and the disproportionate medical and social determinants of health faced by Latino and Asian communities. All partner clinics primarily serve racial/ethnic minorities, including Hispanic/Latino or Asian minorities (83-88%), with >90% below the 200% poverty threshold. Our major FQHCs include the academic UCI Family Health Center, Vietnamese and Korean community Services. We established an FQHC Geriatric Clinic at UCI with the 4 M’s paradigm (i.e., matter, mobility, medication, mentation) integrated into the clinic through the Medicare Annual Wellness Exam. We are disseminating this model to our smaller community sites, along with 4 M presentations. Roughly 5-7% of patients in FQHCs are over 65, allowing leadership time to prepare a foundation for increased growth. Furthermore, we have learned that each FQHC’s cultural setting and infrastructure is unique and requires a customized approach.

